# Interplay Between Gut Microbiota and Amino Acid Metabolism in Heart Failure

**DOI:** 10.3389/fcvm.2021.752241

**Published:** 2021-10-21

**Authors:** Gulinigaer Tuerhongjiang, Manyun Guo, Xiangrui Qiao, Bowen Lou, Chen Wang, Haoyu Wu, Yue Wu, Zuyi Yuan, Jianqing She

**Affiliations:** ^1^Department of Cardiovascular, The First Affiliated Hospital of Xi'an Jiaotong University, Xi'an, China; ^2^Key Laboratory of Environment and Genes Related to Diseases, Ministry of Education, Xi'an, China

**Keywords:** heart failure, gut microbiota, amino acids, metabolism, cardiometabolic disorders

## Abstract

Heart failure (HF) is a complex clinical syndrome of which the incidence is on the rise worldwide. Cardiometabolic disorders are associated with the deterioration of cardiac function and progression of HF. Recently, there has been renewed interest in gut microbiota (GM) and its metabolites in the cardiovascular disease. HF-caused hypoperfusion could increase intestinal permeability, and a “leaky” bowel leads to bacterial translocation and make its metabolites more easily enter the circulation. Considerable evidence shows that the composition of microbiota and amino acids (AAs) has been altered in HF patients, and AAs could serve as a diagnostic and prognostic biomarker in HF. The findings indicate that the gut–amino acid–HF axis may play a key role in the progression of HF. In this paper, we focus on the interrelationship between the AA metabolism and GM alterations during the development of heart failure. We also discuss the potential prognostic and therapeutic value of the gut–amino acid–HF axis in the cortex of HF.

## Introduction

Heart failure (HF) is a complex clinical syndrome and the end-stage of various cardiovascular diseases, which affects approximately 2% of adults worldwide (1). Although there have been great advances in the diagnosis and new treatments of HF, the overall prognosis for patients with HF is still not promising, and the hospital readmission and mortality rates of the patients are still high (2, 3). This makes it particularly important to understand and treat HF more precisely. Nowadays, with the advances of multiomic approaches (e.g., genomics, proteomics, transcriptomic, and metabolomics), novel therapeutic targets and strategies have been emerging to better characterize HF mechanistically as well as to investigate cutting-edge therapeutic strategies (4–7).

A growing number of research findings indicate that gut microbiota (GM) is strongly associated with human health and various diseases, including cardiovascular diseases (8–14). Recent researches have shown the regulating function of the gut microbiota and its metabolites in the manifestation and progression of HF (15, 16). A number of GM-associated metabolites, including short-chain fatty acids (SCFAs), bile acids (BAs), trimethylamine N-oxide (TMAO), amino acids (AAs) metabolites, and so on has been proven to take an active part in HF (16–21). These metabolites are the main pathways through which GM interacts with their hosts. Besides, the recent research have shown that the cardiac proteolysis attenuates in HF (22); thus, amino acids and its metabolites may have a strong impact on host through metabolic pathways. For example, the

tryptophan–kynurenine pathway and glycine-glyR α2-MAPK/ERK pathway have been indicated to obtain a cardioprotective effect (23, 24). However, with the emergence of other metabolite exploration in HF, there is basically no review mainly focused on the relationship among GM, AAs, and HF.

In this review, we reviewed the interrelationship between the long-term GM dysbiosis and AA perturbations in the development of HF. Moreover, we summarized the current researches regarding the alterations in the composition of intestinal microbiota in HF patients and the important pathogenic mechanism of AAs.

## Gut Microbiota Alterations in Heart Failure

HF-caused hypoperfusion could increase intestinal permeability. The “leaky” bowel leads to bacterial translocation, making its metabolites more easily enter the circulation, such as endotoxin (LPS), the trigger of chronic inflammation (16, 25–28). However, except for the mechanism of a “leaky” gut, it is possible that the progression of HF could be regulated by alterations in the composition of GM and its metabolites (16).

Changes in the composition of GM in HF have been confirmed not only in the clinical study but also in experimental animal studies, which helped elucidate the role that microbiota played in the development of HF (14, 15, 29). Table 1 summarizes the several cohort studies that have demonstrated the GM alterations in HF patients (Table 1) (15, 25, 27, 30–33).

**Table 1:** Brief summary of studies investigating heart failure (HF) and alterations in microbiota and amino acids.

**References**	**Sample groups**	**Methods**	**Key findings**
Sandek et al. (27)	22 CHF patients and 22 controls	Biopsies of the sigmoid mucosa taken for fluorescence *in situ* hybridization (FISH)	Mean density of bacteria within mucus was higher in CHF patients; The most frequent strains were Bacteroides/Prevotella in HF patients and controls
			
			
Sandek et al. (30)	65 HF patients and 25 controls	Tested by FISH	CHF patients exhibited increased bacteria restricted to the juxtamucosal zone and a lower intestinal blood flow. The mucosal biofilm was altered in HF patients for higher occurrence of strictly anaerobic *Eubacterium rectal*e group and the strictly anaerobic *Fusobacterium prausnitzii*
			
			
Pasini et al. (25)	60 mild CHF patients, 30 moderate to severe CHF patients, and 20 controls	Microbiota in stool samples was measured after 48 h of incubation. Further proof by using bacterial metabolic tests	CHF patients had massive quantities of pathogenic bacteria and *Candida*, such as *Campylobacter, Shigella, Salmonella, Yersinia enterocolitica*, and *Candida* species
			
			
Luedde et al. (31)	20 HF patients and 20 controls	16s rRNA gene sequencing	HF patients had a lower diversity of the gut microbiota. There was a significant decrease in Coriobacteriaceae, Erysipelotrichaceae, and Ruminococcaceae observed on the family level in HFs. On the genus level, *Collinsella*, uncl. *Erysipelotrichaceae*, and uncl. Ruminococcaceae showed a significant decrease in HF
			
			
Kummen et al. (32)	Two cohorts. Discovery: 40 HFs; validation: 44 HFs; 266 controls	16s rRNA gene sequencing	HF patients had decreased microbial richness and identified changes in 15 taxa, including a depletion of taxa in the Lachnospiraceae family, which are known producers of butyrate
			
			
Beale et al. (33)	26 HFpEFs, 39 metropolitan controls and 28 regional controls	16s rRNA gene sequencing	There was a significant difference in α-diversity and β-diversity between both cohorts of controls and HFpEFs. HFpEFs had a lower Firmicutes-to-Bacteroidetes ratio but not significantly, and depleted bacteria that are short-chain fatty acid producers
			
			
Cheng et al. (34)	51 controls and 183 HFs; validation: 63 controls and 218 HFs with stage C	Untargeted metabolic analysis by LC-MS	A panel of metabolites, including histidine, phenylalanine, spermidine, and phosphatidylcholine C34:4, has a diagnostic value similar to B-type natriuretic peptide (BNP). The prognostic value of the metabolite panel, which consisted of the asymmetric methylarginine/arginine ratio, butyrylcarnitine, spermidine, and the total amount of essential amino acids, was better than that of BNP
Wang et al. (35)	94 controls and 599 acute/decompensated HFs; validation: 391 HFs	Plasma was analyzed by UPLC	High-risk type 1 (leucine ≥145 μM and phenylalanine ≥88.9 μM), high-risk type 2 (leucine <81.2 μM) were associated with higher event rates. The prognostic value of types 1 and 2 remained significant after adjusting for age, BNP, and other risk factors in HF
Wang et al. (36)	890 HF outpatients to assess metabolic status, 387 patients to measure metabolic equivalents (MET).	Plasma samples measured by UPLC	HOP (plasma concentrations of histidine, ornithine, and phenylalanine) scores of ≥8.8 stratified patients at higher risk of composite events in a variety of HF populations. In multivariable analysis, HOP scores ≥8.8 remained a powerful event predictor, independent of other risk factors
Chen et al. (37)	115 HFs and 37 controls	Plasma samples measured by UPLC	Phenylalanine ≥112 μM was associated with a lower accumulative survival rate and predicted death over 1 year independently
Lu et al. (24)	C57BL/6J mice and male SD rats	Cardiac hypertrophy and HF were induced by TAC surgery or Ang II	Glycine may be a novel cardioprotector against pressure overload-induced cardiac hypertrophy; the protection of glycine might be mediated by glyR α2 through the MAPK (JNK, ERK1/2, and p38) signaling pathways
Rozentryt et al. (38)	Placebo group:6; nutrition group:23	Intervention includes additional 600 kcal per day (proteins 20 g, carbohydrates 72 g, fat 26 g)	The feasibility of oral nutritional supplement in cachectic patients with heart failure and significant clinical benefit in terms of body size and body composition, laboratory parameters, and quality of life
Wu et al. (39)	Placebo group:12; active group:14	Intervention includes a combination of 8 g/day of L-alanyl-L-glutamine and 6.5 g/day of fish oil	The combined supplementation of L-alanyl-L-glutamine and PUFA did not improve exercise performance or muscle function but increased lean body mass and quality-of-life in patients with chronic stable HF
Pineda-Juárez et al. (40)	26 controls and 29 experimental group	Experimental group: the resistance exercise program and received 10 g/day BCAA supplementation, control group: the resistance exercise program.	Improvements in physical and functional capacities are attributed to resistance exercise program but not to the BCAA supplementation

The study of Sandek et al. might play a fundamental role in the exploration of the relationship between HF and microbiota (27, 30). They found that the mean density of microbiota within mucus was higher in CHF patients, and the mucosal biofilm was altered for a higher occurrence of strictly anaerobic *Eubacterium rectale* group and the strictly anaerobic *Fusobacterium prausnitzii*. The first study using 16s rRNA gene sequencing approach was reported by Luedde et al. in 2017 and revealed that HF patients had a decreased diversity of the microbiota in parallel with the downregulation of vital microbiota groups (31). More recently, Kummen et al. identified microbiota changes in 15 taxa, including depletion of taxa in the *Lachnospiraceae* family, which are known producers of butyrate (32). Another study showed that HFpEFs had a lower *Firmicutes*-to-*Bacteroidetes* ratio but not significantly and depleted bacteria that are short-chain fatty acid producers (33). Additionally, there has been evidence that *Bacteroides fragilis* and *Ruminococcus* had a positive effect on HF (41).

Nonetheless, current studies have a controversy on the shifting tendency of the specific microbiota and its function in HF (14). Further studies are required to explore the link between HF and microbiota and to investigate the exact effects of microbiota.

## Bacterial Metabolism of Amino Acids

As an essential part of nutrients in the diet, AAs could affect the nutrition of the host by interacting with gut microbiota. In addition, AAs also play a vital role in regulating the diversity and abundance of AA-fermenting microbiota for their heterogeneity in turn (42–46).

The catabolism of protein in the diet is inseparable from the GM. GM promotes the proteolysis of protein through host- and bacteria-derived proteases and peptidases, which are used for incorporation of amino acids into structural and respective proteins. Besides, GM facilitates protein fermentation, the productions of which are SCFAs, gases (H_2_, CO_2_, CH_4_, andH_2_S), nitrogenous metabolites, amines, indoles, and so on. There are several ways of disposal of the AA production: (1) excretion by feces or breath, (2) utilization by microbiota, (3) detoxification by colonic epithelium, and (4) absorption by intestinal epithelium and entering circulation (42).

AA-fermenting microbiota are effectively distinct in the intestine for the preference of AA utilization. Preferred AA substrates of *Clostridium* genus bacteria are lysine or proline, and the *Peptostreptococcaceae* genus played a vital role in glutamate or tryptophan utilization (42, 47). Anaerobes ferment aromatic amino acids, including *Bacteroides, Lactobacillus, Bifidobacterium, Clostridium*, and *Peptostreptococcus* (43). Moreover, it is shown that *Clostridium sporogenes* using aromatic amino acids as substrates generated 12 compounds, one of which, indolepropionic acid (IPA), act as a major metabolite and could affect intestinal permeability and immunity (48). *Lactobacillus* could also use tryptophan to produce indole metabolites offering mucosal protection from inflammation (49).

Moreover, it is noteworthy that several species are the key driver of AA metabolism, including *Staphylococcus aureus, Megasphaera elsdenii, Bacteroides* spp. et al. (42, 47). In conclusion, AAs and microbiota have a strong interaction with each other, and microbiota contributes to host AA homeostasis.

## Dysregulated Amino Acid Metabolism in Heart Failure

As metabolomics is maturing, it is being utilized for early identification of organ function and dysfunction, and exploration of original disease pathways (7, 50). It is becoming clear that circulating metabolite profiles have a strong connection with HF severity and prognosis, which serve as promising novel biomarkers for identifying HF progression. Additionally, circulating amino acid profiles can directly reflect the host nutrition status, including food intake, absorption, tissue synthesis, and breakdown. Recent studies have provided evidence for microbial metabolism of AA in HF and investigated the value of AA for the identification and evaluation of HF.

Branched-chain amino acids (BCAAs), including valine, leucine, and isoleucine, are the major components in most mammals and account for nearly 35% of essential amino acids and 18% of the total amino acids (51, 52). One of the pivotal synthesis pathways of BCAA is in the microbiota, and uptake of BCAA from food is indispensable in humans. Different from not being synthesized in humans, BCAA catabolism not only occurs in the microbiota but also in humans and is mainly absorbed in the gut (52). More attention has been paid to the association between elevated circulating BCAA and HF (53–55). There are considerable evidence convincing the alterations of BCAA during HF, but these studies fail to explain the underlying mechanisms of how BCAA contributes to the progression of HF.

Large-scale trails, to date, have been carried out to demonstrate the relationship between circulating phenylalanine concentrations and HF (34, 35, 37, 56–59). Most of them suggest that increased phenylalanine could provide both diagnostic and prognostic value for HF. Consistent with such changings, there was a paralleling increasing trend in the serum tyrosine levels (35). Phenylalanine and tyrosine participates in the biopterin cycle; accumulation of them is a signal for tetrahydrobiopterin depletion, which often causes a problem with the production of NO and leading to cardiac dysfunction (34, 37). What is more, higher phenylalanine levels were correlated with higher C-reactive protein levels and higher pro-inflammation (37). It is an indication that HF patients with high phenylalanine levels have a more severe inflammation.

Tryptophan is one of the aromatic amino acids, and the kynurenine pathway (KP) is the key mechanism for tryptophan degradation (60–62). It has been shown that kynurenine has a strong relation to the pathophysiology of HF, such as inflammation, apoptosis, and oxidative stress (62). Increasing plasma levels of the kynurenine metabolites and the kynurenine:tryptophan ratio were associated with increased mortality in HF patients (23, 62). Furthermore, tryptophan is a pivotal substance for serotonin (5-HT) synthesis, and more than 90% of serotonin is synthesized from tryptophan in the intestine (61). Prior studies convincingly showed the considerable changes of 5-HT and 5-HT(2A)R in heart failure mice, and 5-HT2B receptor antagonists could inhibit right ventricular fibrosis through reducing collagen deposition (63–65). The newest study conducted by Cristina Razquin et al. examined the serotonin pathway and tryptophan-indole-3-propionic acid pathway of tryptophan metabolism, providing more evidence for understanding the relationship between tryptophan-related metabolites and HF (23).

In the pathophysiology of HF, failing hearts suffer from mitochondrial dysfunction, and their energy supplementation shifts from fatty acids to glucose utilization (34, 66). Histidine, arginine, and glutamine can be transferred into glutamate, offering energy and ornithine for cardiac tissues by participating into the Krebs cycle as alpha-ketoglutarate or the glutamate–ornithine–proline pathway (34). Previous studies further support the implicated mechanism of metabolic pathways in HF pathogenesis. It is almost certain that HF patients have a commonly decreasing trend in histidine, arginine, glutamine, and a reversely increase in ornithine (34). Furthermore, glutamate metabolism is ranked in the top 50 pathways in the enrichment overview for plasma in HF mice and patients (67).

As a nonessential amino acid, glycine has been repeatedly reported to have a hand in anti-inflammatory response, growth, and cytoprotection (68, 69). Recently, its beneficial effect in antihypertrophy and HF has been reported. Glycine antagonized left ventricular (LV) hypertrophy and cardiac fibrosis in HF mice (24). This paved a path for the generation of a novel treatment of HF.

In summary, a considerable number of previous studies confirmed that there commonly exists a disorder of AAs with a different trending in HF. Consistent with that, these results further supported the connection between HF and AAs.

## Amino Acid As a Diagnostic and Prognostic Marker for Heart Failure

AAs have been shown to be a potential promising biomarker with significant diagnostic and prognostic values for HF. What we know about the values of AAs is largely based on the clinical studies that evaluate the possibility and stability of AAs as original biomarkers.

The study finished by Cheng et al. is one of the most influential researches to assess the metabolites and has identified a panel of four metabolites (histidine, phenylalanine, phosphatidylcholine diacyl C34:4, spermidine), which provided a similar diagnosis value and a better prognostic value than the conventional biomarker, BNP (34).

A recent study has classified patients at high risk for HF-related events into two groups: high-risk type 1 (leucine ≥145 μM and phenylalanine ≥88.9 μM), high-risk type 2 (leucine <81.2 μM), and other HF patients were placed into the low-risk group (35). Compared with the low-risk type, types of high-risk patients had a lower event-free survival rate, especially type 2 high-risk patients, which were characterized by severe malnutrition. This kind of novel simplified amino acid-based risk stratification offered a prognostic value for HF patients.

Wang et al. have assessed amino acid-based profile including histidine, ornithine, and phenylalanine (HOP score) (36). They have found a strong association between HOP score and cardiac function evaluated by the 6-min walking distance, which can compensate for the limitation of the NYHA Functional Classification System. A HOP score of ≥8.8 was associated with more risk factors for HF events.

In a study conducted by Chen et al., they measured the prognostic mortality value of phenylalanine in HF patients (37). Phenylalanine level has appeared to be positively related to the mortality of HF patients. In contrast to HF patients with phenylalanine <112 μM, patients with phenylalanine >112 μM had higher APACHE II and SOFA scores with higher mortality. Their analysis demonstrated that phenylalanine was an independent predictor of mortality and suggested setting 112 μM as a critical point for identifying different outcomes.

Up to now, there have been considerable clinical researches published to describe the significant value of AAs as diagnostic and prognostic biomarkers in HF. These results indicate that dysregulated AA is not only the result of HF but also a potential indicator for the progression of HF.

## Gut–Amino Acid–Heart Failure Axis As a Potential Therapeutic Target for Heart Failure

A burgeoning number of relevant studies have demonstrated that there is a microbiota dysbiosis and amino acid alterations in HF; otherwise, there is a tight connection among gut microbiota, amino acids, and heart failure. What stands out is the promising values of the gut–amino acid–HF axis as a potential therapeutic target for HF.

Probiotics are live microorganisms, and prebiotics are nondigestible food products, which can change the microbiota composition and activity (14). Both of them are reported to be beneficial to the host and be cardioprotective. *Lactobacillus rhamnosus* GR-1 has been considered as a potential therapy for the attenuation of heart failure, so did *Lactobacillus plantarum 299v* and *Saccharomyces boulardii* (70, 71). However, antibiotics regulate the composition of microbiota through inhibiting specific types of negative bacteria (72). Vancomycin could improve the cardiac function in rats; polymyxin B had a similar function (70, 73). At the same time, it also increases the possibility of drug-resistant microbiota (74).

Dietary intervention for delaying the progression of HF mainly follows the guidelines from the American College of Cardiology/American Heart Association (75). The recommended Dietary Approaches to Stop Hypertension (DASH) eating plan has been assessed by several clinical trials and suggested its beneficial role for reducing HF incidence (76–79). It is also reported that several nutritional factors could change intestinal permeability, acting as a potential dietary intervention of HF (80).

Fecal microbiota transplant (FMT) is a budding way of transferring a positive microbiota into the unhealthy receiver. It has been proven to be effective in the treatment of *Clostridium difficile* infection, inflammatory bowel disease, and metabolic syndrome (81, 82). However, it also brings about a problem of virus transmission (83). Up to now, there are no related trials in HF.

Even though considerable studies have demonstrated that quite a few amino acids are useful for HF outcomes, there are some studies that evaluate how effective the amino acids are for HF treatment. There is one RCT study indicating that high-caloric protein-rich oral supplement has significant clinical benefits in terms of body composition and quality of life in CHF (38). Another RCT shows that supplementation of L-alanyl-L-glutamine and PUFA have the same effects in the CHF patients (39). However, BCAA supplementation has no improvements on physical and functional capacities in patients with HF (40). In one experimental study, combing β-alanine and histidine with exercise could elevate functional capacity and maximum strength in rats with CHF (84). Although it has been proven that glycine could reduce cardiac fibrosis in HF mice, its cardioprotective role remains to be validated in clinical trials.

## Conclusions

**Figure 1:**
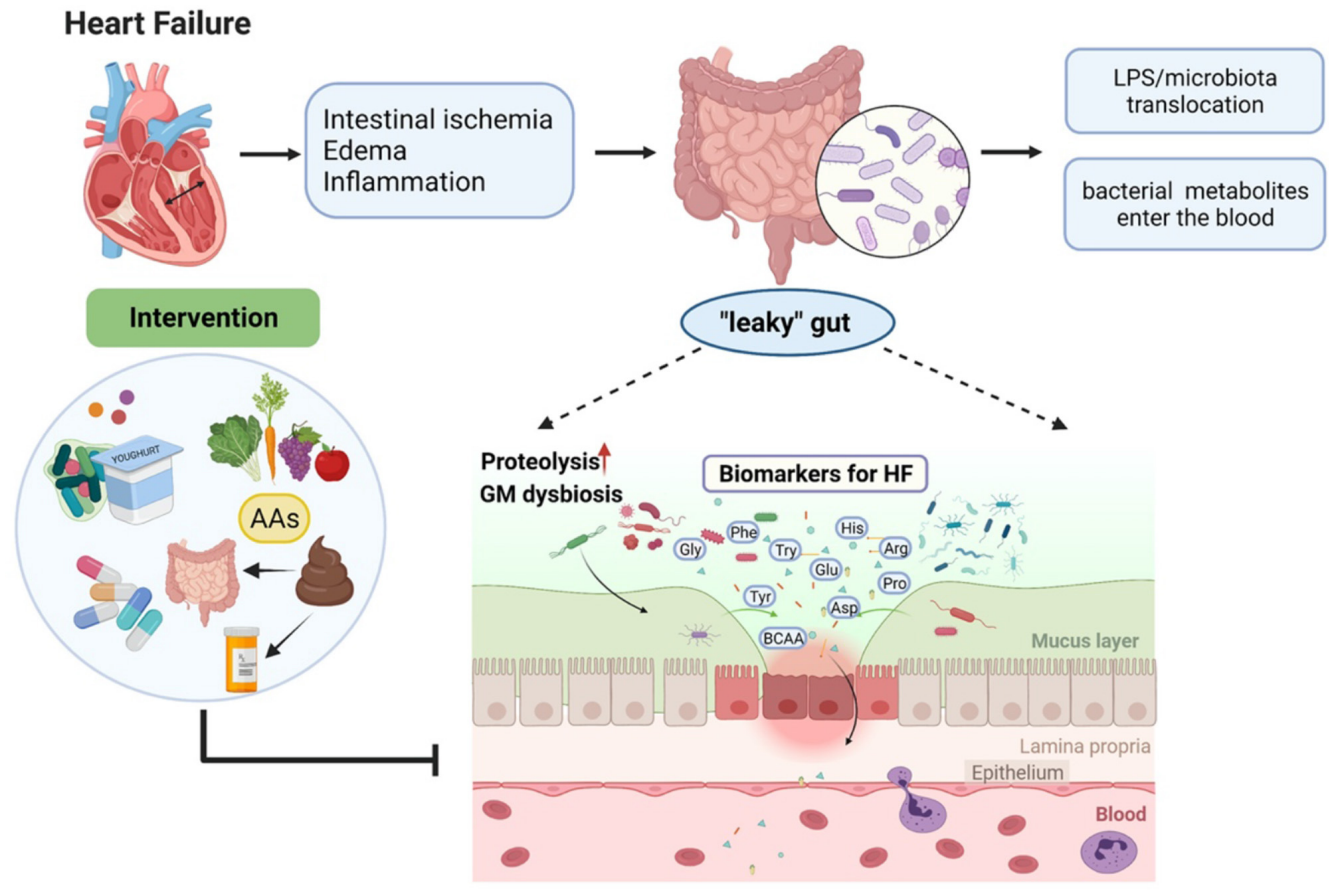
Heart failure (HF)-caused gut microbiota dysbiosis leads to metabolite disorders and therapeutic interventions.

Overall, current clinical studies have demonstrated the connection among gut microbiota, the circulating amino acids, and HF, the graphic abstract is shown in Figure 1. A number of AAs have been proven to serve as a diagnostic and prognostic biomarker in HF. Besides, several AAs are tested to show the positive effects of improving cardiac function in HF. Nevertheless, these results may not be adequate to elucidate the cause and effect between gut microbiota and HF. Further mechanism studies and clinical trials are needed to evaluate the gut–amino acid–HF axis as a potential therapeutic target for HF and to develop a deeper understanding of this axis in the future.

This review has pivotally presented the evidence of the gut–amino acid–HF axis as a whole and contributed in several ways for our understanding of HF. However, these findings and conclusions are limited by the quality and quantity of the relative research. Therefore, it is recommended that further research should be undertaken deeply in this axis.

## Author Contributions

GT, ZY, and YW designed and executed the review. GT, MG, XQ, BL, CW, HW, and JS drafted the manuscript, figure, and table. All authors contributed to the article and approved the submitted version.

## Funding

This work was supported by the National Natural Science Foundation of China (81800390).

## Conflict of Interest

The authors declare that the research was conducted in the absence of any commercial or financial relationships that could be construed as a potential conflict of interest.

## Publisher's Note

All claims expressed in this article are solely those of the authors and do not necessarily represent those of their affiliated organizations, or those of the publisher, the editors and the reviewers. Any product that may be evaluated in this article, or claim that may be made by its manufacturer, is not guaranteed or endorsed by the publisher.

## Abbreviations

HF: heart failure
GM: gut microbiota
SCFA: short-chain fatty acid
BA: bile acid
TMAO: trimethylamine N-oxide
AA: amino acid
IPA: indolepropionic acid
BCAA: branched-chain amino acid
KP: kynurenine pathway
5-HT: serotonin
LV: left ventricular
DASH: Dietary Approaches to Stop Hypertension
FMT: fecal microbiota transplant.
